# Solving Energy-Aware Real-Time Tasks Scheduling Problem with Shuffled Frog Leaping Algorithm on Heterogeneous Platforms

**DOI:** 10.3390/s150613778

**Published:** 2015-06-11

**Authors:** Weizhe Zhang, Enci Bai, Hui He, Albert M.K. Cheng

**Affiliations:** 1 School of Computer Science and Technology, Harbin Institute of Technology, Harbin 150001, China; E-Mails: encibai@hit.edu.cn (E.B.); hehui@hit.edu.cn (H.H.); 2 Department of Computer Science, University of Houston, Houston, TX 77004, USA; E-Mail: cheng@cs.uh.edu

**Keywords:** energy-aware scheduling, real-time tasks, heterogeneous multiprocessor systems, shuffled frog leaping algorithm

## Abstract

Reducing energy consumption is becoming very important in order to keep battery life and lower overall operational costs for heterogeneous real-time multiprocessor systems. In this paper, we first formulate this as a combinatorial optimization problem. Then, a successful meta-heuristic, called Shuffled Frog Leaping Algorithm (SFLA) is proposed to reduce the energy consumption. Precocity remission and local optimal avoidance techniques are proposed to avoid the precocity and improve the solution quality. Convergence acceleration significantly reduces the search time. Experimental results show that the SFLA-based energy-aware meta-heuristic uses 30% less energy than the Ant Colony Optimization (ACO) algorithm, and 60% less energy than the Genetic Algorithm (GA) algorithm. Remarkably, the running time of the SFLA-based meta-heuristic is 20 and 200 times less than ACO and GA, respectively, for finding the optimal solution.

## 1. Introduction

Multiprocessor systems can achieve higher performance by thread-level parallelism with a lower clock frequency, which have advantages over highly superscalar uniprocessor architectures [1]. Multiprocessor systems can be classified into homogeneous and heterogeneous ones. The use of Heterogeneous Multiprocessor Systems (HMS) can sometimes dramatically improve performance and energy consumption relative to homogeneous ones [2]. More and more real-time applications are ported to HMS, such as gaming for Xbox360 and aerospace applications for QorIQ from FreeScale [3,4].

Task scheduling is challenging and complex for real-time applications especially on HMS. Recently, reducing energy consumption is becoming more and more important in order to lower cooling requirement and overall operational costs. Also, minimizing the energy consumption can help to prolong the battery lifetime for battery-driven embedded systems and guarantee the real-time constraints. Thus, there is a need to shift the focus from solely optimizing multiprocessor resource management without violating task deadlines to optimizing them for energy efficiency.

The problem of scheduling independent, periodic real-time tasks on HMS without energy consideration is already NP-hard in the strong case [5]. The additional objective for saving energy further complicates the problem.

A classical solution for energy-aware real-time task scheduling is to formulate it as an Integer Linear Programming (ILP) problem [6,7]. Then, a linear relaxation heuristic is introduced to solve the equivalent ILP problem. Besides, some approximate algorithms are proposed in [8,9], which are (*m* + 2)-approximation and 0.5-approximation, respectively, where *m* is the number of the available processor types. A dynamic programming method is also proposed to provide certain worst-case performance guarantee [10]. However, these approximate or heuristic algorithms cannot efficiently work in practice for large size problems, although these studies enlighten the way. Recently, swarm intelligence algorithms such as Ant Colony Optimization (ACO) and Genetic Algorithm (GA) heuristics [11] are introduced. Comparing to the ILP and approximation algorithms, ACO and GA are more efficient while they are still time-consuming for large-scale problems. Thus, new quick meta-heuristics for energy-aware real time scheduling need further research.

A meta-heuristic designates a computational method that optimizes a problem by iteratively trying to improve a candidate solution with regard to a given measure of quality. A particularly successful meta-heuristic, the Shuffled Frog Leaping Algorithm (SFLA) [12,13] is inspired by the behavior of the evolution of frog foraging. The frogs are divided into subgroups named as memeplexes, which have different cultures. Each memeplex performs a local search. The frogs in each memeplex can exchange ideas. After several evolution steps, memeplexes exchange the ideas in a shuffled process. Until the convergence criteria are satisfied, the algorithm does not stop. In essence, SFLA combines the benefits of Particle Swarm Optimization (PSO) and Memetic Algorithm (MA) for mixing information from parallel local searches to move toward a global solution.

The main objective of this work is to design novel real-time scheduling algorithms based on SFLA, which can quickly find an optimal solution not only satisfying hard task deadlines but also reducing energy consumption.

Specifically, our work aims to:

- Formulate the energy-aware real-time taskscheduling problem for HMS by incorporating the energy consumption into the constraints and optimization objectives.
- Develop SFLA-based energy-aware real-time scheduling algorithms that improve the energy efficiency of a real-time system without violating the task deadlines under a short decision time.
- Perform extensive experiments comparing our approach with traditional and other swarm intelligence algorithms.

The rest of the paper is organized as follows. Section 2 discusses related work, followed by the formulation of the energy-aware real-time scheduling problem for HMS presented in Section 3. Section 4 describes the overview and prototype of the shuffled frog leaping algorithm. The proposed energy-aware real-time scheduling algorithms based on SFLA are discussed in Section 5. A performance analysis of the proposed energy-aware task scheduling algorithms is presented in Section 6. Section 7 concludes the paper with summary and future research directions.

## 2. Related Works

Real-time scheduling algorithms and schedulability analyses have gained extensive studies for uniprocessor and homogeneous multiprocessor systems [14,15]. In this section, we discuss related works mainly focusing on heterogeneous multiprocessor systems (HMS) for real-time tasks, which are the most relevant to our research. First, we survey research results of real-time scheduling on HMS without considering energy consumption. Then, existing studies of energy-aware real-time scheduling on HMS are summarized.

### 2.1. Real-Time Scheduling on HMS

We classify existing real-time scheduling studies for HMS into two categories based on task dependencies, namely, *independent tasks* and *tasks with precedence constraints*.

*Independent tasks* do not have any control or data dependency. In 2004, to our best knowledge, Baruah first proves that the real-time scheduling problem of independent periodic tasks on HMS is NP-hard in the strong case [5]. Also, [16,17] model the same problem as an Integer Linear Programming (ILP) problem. Polynomial time-complexity can be attained by the relaxation of the ILP formulation to Linear Programming (LP) although neither attains linear polynomial time-complexity. Thus, many combinatorial optimization algorithms are applied to this problem, which can lead to good solutions in a comparatively short time. Chen *et al.* [18] propose an Ant Colony Optimization (ACO) heuristic and a Genetic Algorithm (GA) heuristic to solve the problem. Extensive experiments prove that the ACO heuristic has better performance than the GA heuristic and the LP approximation algorithm in [16,17].

The task model in [19,20] is more complicated, which considers the tasks’ *divisibility*. A *divisible* task means that its load can be partitioned into an arbitrarily large number of load fractions. Based on *divisible load theory* (DLT) [21], a DLT and (Earliest Deadline First) EDF mixed scheduling approach is proposed. On the contrary, recent works in [22,23] argue that the types of HMS should be simplified as two unrelated types of processors because most HMS only have two types of processors.

*Tasks with precedence constraints* are often described by a task graph, more formally a weighted Directed Acyclic Graph (DAG). DAG scheduling is usually based on *list scheduling*, *clustering scheduling* and *task duplication* heuristics. The *list scheduling* algorithm for real-time task mapping on HMS includes the task selection phase and the processor selection phase [24,25]. Tasks are scheduled based on their priorities. The *clustering scheduling* algorithm maps nodes of the DAG onto labeled clusters. All tasks of the same cluster run in the same processor [26]. The *task duplication* algorithm uses selective task duplication to increase the guarantee ratio of real-time tasks. Auluck *et al.* [27] propose a scalable duplication-based algorithm for scheduling real-time tasks with precedence constraints on HMS. Recently, several swarm intelligence algorithms such as genetic algorithms are introduced. Yoo *et al.* [28,29] combine a simulated annealing (SA) and a multi-objective genetic algorithm to minimize the tardiness and completion time for real-time tasks.

Our research in this paper shares the same real-time task model (independent, periodic task with hard deadlines) and processor model (preemptive, heterogeneous multiprocessor) with the related works in [5,16,17,18]. However, the key difference is that the above studies [5,16,17,18,19,20,21,22,23,24,25,26,27,28,29] do not consider energy consumption while our work not only guarantees the satisfaction of the task deadlines but also reduces their energy consumption.

### 2.2. Energy-Aware Real-Time Scheduling on HMS

Energy-aware real-time scheduling can be classified into two categories: (1) *Dynamic Voltage* and *Frequency Scaling* (*DVFS-based*), which allows processors to run at different energy levels by adjusting the voltage or clock frequency; and (2) *Non-DVFS-based* means that processors are not equipped with the DVFS technique. Chen *et al.* [30] summarize the non-DVFS research on energy-aware real-time scheduling. Li *et al.* [31] survey the DVFS-based energy-aware real-time scheduling on *homogeneous* multiprocessor systems. Thus, we only focus on recent advances in DVFS-based energy-aware real-time scheduling on HMS, which are closest to our research.

*Independent tasks*: Yu *et al.* [6] formulate the energy-aware real-time scheduling on HMS as an ILP problem. A linearization heuristic (LR-heuristic) is proposed to minimize the energy consumption and guarantee the satisfaction of the task deadlines. Chen *et al.* [8] introduce a (*m* + 2)-approximation polynomial-time algorithm tackling the same multiprocessor allocation problem for energy-constrained real-time scheduling. Hung *et al.* [9] present a 0.5-approximation algorithm by allowing the non-DVFS processors to be involved. Based on partition scheduling, a dynamic programming method with state pruning [10] and an approximation algorithm based on ILP relaxation [7] provide fully polynomial-time approximation schema to schedule the energy-aware tasks. More recently, swarm intelligence algorithms such as ACO and GA [11] are introduced to save energy for the same task and processor models.

*Tasks with precedence constraints*: Related studies are relatively rare for energy-aware real-time scheduling with precedence constraints on HMS. Schmitz *et al.* [32] introduce the genetic list scheduling and a mapping algorithm to reduce the energy consumption. Luo *et al.* [33] propose a combined static and dynamic algorithm for ensuring soft aperiodic tasks’ service quality and energy consumption reduction.

Our research in this paper has similar task, processor and energy models (DVFS-based) as in the related works [6,7,8,9,10,11]. Similarly, minimizing the energy consumption and guaranteeing the task deadlines are also our optimization objectives. However, the approximation algorithms or heuristics in [6,7,8,9,10,11] are time-consuming and difficult to work in practice. Our shuffled frog leaping algorithm not only can obtain many feasible solutions in a shorter time but also save more energy than the related works in [6,7,8,9,10,11].

## 3. Formulation

This section first provides the models of the real-time tasks and HMS to ease further discussion. After that, a DVFS-enabled energy model is given. Then, the energy-aware real-time tasks scheduling problem and its linear programming formulation are presented. Finally, we prove the equivalency of this problem and its ILP formulation.

### 3.1. Real-Time Task and Processor Models

Let *T* = {*T*_1_, *T*_2_, ..., *T_n_*} denote a real-time task set with *n* tasks *T_i_* (*i* = 1, 2, ..., *n*). Each task *T_i_* is independent, periodic and indivisible with hard deadlines. A real-time task *T_i_* is described by a 4-tuple (*a_i_*, *c_i,j_*, *d_i_*, *p_i_*), where *a_i_* denotes its initial arrival time, *c_i,j_* denotes the worst case execution time (WCET) of *T_i_* when it is assigned to processor *M_j_*, *d_i_* denotes its relative deadline, and *p_i_* denotes its arrival interval between two consecutive instances of the task. We assume that all of the tasks are released at the same time 0, which means *a_i_* = 0. The deadlines of tasks are considered to be *implicit*, which means the relative deadline of task *T_i_* is assumed to be equal to its period, *i.e.*, *d_i_* = *p_i_*. Each task can only be assigned to a unique processor and task migration is forbidden. After the task, *T_i_* is assigned to a processor, we simply use the earliest-deadline-first (EDF) policy for task scheduling because it is an optimal uniprocessor scheduling policy for independent real-time tasks.

Let *M* = {*M*_1_, *M*_2_, …, *M_k_*} denote a heterogeneous platform, where each *M_j_* (*j* = 1, 2, …, *k*) is a preemptive processor. *M_j_* executes only one instruction in a clock cycle. We use *f_j_* to denote the clock frequency and *c_i,j_* to denote the running time for *T_i_* on processor *M_j_*. If we define *cycle_i_* as the number of clock cycles for *T_i_*, *c_i,j_* and *f_j_* can be formulated as follows: *c_i,j_* = *cycle_i_*/*f_j_*. The utilization of task *T_i_* assigned to processor *M_j_* is denoted by *u_i,j_*, which is a real number in (0, 1)∪+∞. If *c_i,j_* > *d_i_*, we set *u_i,j_* to +∞, which means *T_i_* cannot execute on *M_j_*. Otherwise, *u_i,j_* = *c_i,j_*/*d_i_*. Let the utility matrix *U_n×k_* denote the computing costs when *n* tasks run on *k* processors. Each element *u_i,j_* in *U_n×k_* indicates the computing costs when *T_i_* runs on *M_j_*.

Let matrix *X_n×k_* denote a scheduling (mapping) of *n* tasks to *k* processors. Each element *x_i,j_* in the matrix denotes whether *T_i_* is assigned to *M_j_*. As the tasks are indivisible, each *x_i,j_* has only two kinds of values, either 0 or 1. *x_i,j_* = 1 indicates that *T_i_* is assigned to *M_j_* and *x_i,j_* = 0 indicates that *T_i_* is not assigned to *M_j_*.

### 3.2. Energy Model

The HMS is DVFS-enabled, where processors can run at different power settings. The energy consumption of a processor is mainly contributed by the dynamic energy consumption resulting from the charging and discharging of gates on the DVS CMOS circuits [8].

Let *E_i,j_* denote the energy consumption of *T_i_* on *M_j_* in one period. The energy consumption accounts for the energy consumption of the CPUs, which dominates the overall energy consumption including the memory access, I/O, *etc*. It can be formulated as follows:

(1) $$E_{i,j}=Power_{i,j}\times c_{i,j}\approx (C_{ef}\times \frac{f_{j}^{3}}{l^{2}})\times c_{i,j}=\frac{C_{ef}}{l^{2}}\times cycle_{i}\times f_{j}^{2}$$

where *C_ef_* and *l* are constants [34,35].

Let *Energy* denote the total energy consumption of *n* tasks on *k* processors, which is formulated as follows:

(2) $$Energy=\sum_{i=1}^{n}\sum_{j=1}^{k}E_{i,j}\times x_{i,j}$$

Let *Max_Energy* denote the theoretical maximum energy consumption of *n* tasks on *k* processors, formulated as follows:

(3) $$Max\_Energy=\sum_{i=1}^{n}\overset{k}{\underset{j=1}{MAX}}(E_{i,j})$$

Note that the tasks have different periods, their total energy consumption is calculated based on the least common multiple of periods of all tasks, denoted by LCM.

### 3.3. Problem Definition and Formulation

**Problem Definition:** We have the following Energy-aware Real-time Tasks Scheduling Problem (*e-RTSP*) for HMS. The objective of the energy-aware real-time scheduling is to minimize the energy consumption by partitioning the task set *T* of *n* tasks into several disjoint subsets, in which all the tasks in a partition of tasks are executed on an allocated processor in *M*. Meanwhile, the execution time of the tasks should satisfy the constraints of their hard deadlines and the cumulative computing consumption should be under the computing utility bound of each processor. A feasible solution is optimal if its energy consumption is minimal among all feasible solutions.

The *e-RTSP* problem can be formulated as an integer linear programming problem as follows:

**Definition 1. *ILP formulation for e-RTSP***: *Given T = {T_1_, T_2_, …, T_n_} and M = {M_1_, M _2_, …, M_k_}, find the optimal scheduling X_n×k_ to minimize Energy, which is subject to the following constraints:*

(1) *x_i,j_* *is either 0 or 1, (i=1,2,…,n; j=1,2,…,k)*
(2) $\forall i\;\sum_{j=1}^{k}x_{i,j}=1$, *(i=1,2,…,n; j=1,2,…,k)*
(3) $\forall j\;\sum_{i=1}^{n}x_{i,j}\times u_{i,j}\leq U$, *(i=1,2,…,n; j=1,2,…,k)*
(4) *e_i,j_ ≤ d_i_*, *(i=1,2,…,n; j=1,2,…,k)*

*where U is the maximum computing capacity that each processor can undertake. We set U = 1 without loss of generality.*

There are four constraints above. The first one defines the *x_i,j_* = 1 or 0 when the task *i* is assigned to processor *j* or not, respectively. The second constraint ensures every task should be assigned to one and only one processor. The third one ensures that the total computing consumption of processor *M_j_* should not overtake the maximum computing capacity *U*. The last constraint ensures that the hard deadline of the real-time tasks can be guaranteed.

### 3.4. Equivalency of e-RTSP and Its ILP Formulation

The problem of *e-RTSP* is formulated as an ILP problem. In this section, we will prove (Theorem 1) the equivalency of the two problems.

**Lemma 1.** *If the ILP problem in Definition 1 has a feasible solution when U = 1, then the feasible solution is mapped to a feasible schedule of the e-RTSP problem.*

**Proof.** If the linear programming problem in Definition 1 has a feasible solution when *U* = 1, then every constraint is satisfied. Constraints (1) and (2) mean that each task in *T* = {*T*_1_, *T_2_*, ..., *T_n_*} is assigned to a processor completely without decomposition and migration, which correspond to the restricted conditions in our task scheduling model. Constraint (4) indicates that the hard deadline of each task *T_i_* must be met on the heterogeneous platform. Constraint (3) means that for each processor *M_j_* (*j* = 1, 2, …, *k*) we have $\sum_{i=1}^{n}x_{i,j}\times u_{i,j}\leq 1$. Therefore, if a task set {*T_j_*_1_,*T_j_*_2_, ..., *T_jl_*} is assigned to *M_j_*, then $\sum_{i=1}^{l}c_{j_{i},j}/d_{i}\leq 1$ can be set up. If a uniprocessor has $\sum_{i=1}^{l}c_{j_{i},j}/d_{i}\leq 1$, a proper scheduling algorithm can find a feasible schedule, which meets the deadlines of all the periodic tasks’ instances, which is proven in [36]. We complete the proof of Lemma 1. □

**Lemma 2.** *If the e-RTSP problem has a feasible schedule, then the ILP problem in Definition 1 has a feasible solution when U = 1.*

**Proof.** According to the task model, processor model, scheduling model and utility model described earlier, if the problem of assigning real-time tasks onto HMS has a feasible schedule, it is obvious that the constraints (1), (2) and (4) in Definition 1 are satisfied. Next, we will show that the constraint (3) is satisfied as well. It is assumed that every instance of each task arrives instantly.

For a feasible schedule, a uniprocessor *P_j_* is selected, and it is assumed that *h* tasks *T_i_* (*i* = 1, 2, ..., *h*) are assigned to *M_j_* in the scheme, without loss of generality. For all these *h* tasks *T_i_* (*i* = 1, 2, ..., *h*), let *D* denote the least common multiple of their periods. Let *n_i_* denote the number of the instances of each task *T_i_* (*i* = 1, 2, ..., *h*) arriving in the time interval [0, *D*). Since the schedule is feasible, it can be seen that the total execution time is not more than *D*, namely $\sum_{i=1}^{h}n_{i}\times c_{i,j}\leq D$. Because we have *n_i_ × d_i_ = D* for each task *T_i_* (*i* = 1, 2, ..., *h*), so the following derivation is tenable and the constraint (3) in Definition 1 is satisfied. $\sum_{i=1}^{h}n_{i}\times c_{i,j}\leq n_{i}\times d_{i}(i=1,...,h)\Leftrightarrow \sum_{i=1}^{h}\frac{c_{i,j}}{d_{i}}\leq 1\Leftrightarrow \sum_{i=1}^{n}x_{i,j}\times u_{i,j}\leq 1$ We complete the proof of Lemma 2. □

**Theorem 1**. *The e-RTSP problem is equivalent to its ILP problem in Definition 1*.

**Proof**. Lemma 1 and Lemma 2 have proved the necessity and sufficiency. Thus, Theorem 1 is proven. □

## 4. Overview of Shuffled Frog Leaping Algorithm

The Shuffled Frog Leaping Algorithm (SFLA) was proposed by Eusuff *et al*. in 2003 [12]. SFLA is a novel meta-heuristic algorithm for combinatorial optimization problems. It paves a new way to solve the energy-aware real-time task scheduling problems.

**Figure 1:**
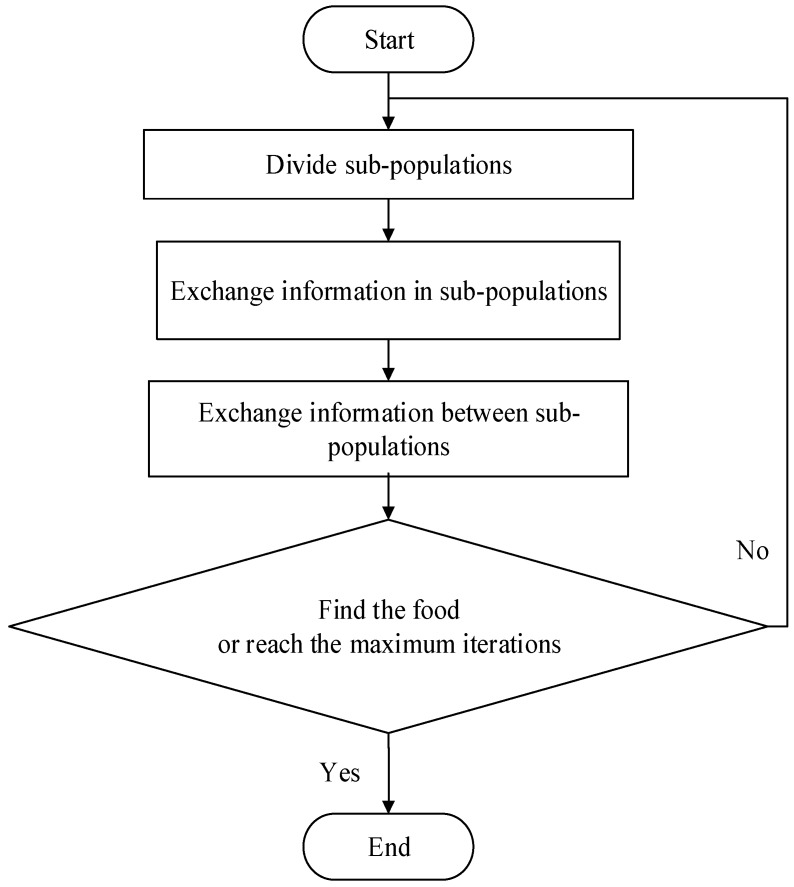
Flow chart of shuffled frog leaping algorithm.

The basic idea of SFLA is inspired by the frog foraging behavior. A lot of frogs live in the wetlands where many stones are discretely placed. The frogs try to find a place with more food by jumping to different stones. Every frog has its own culture, and the frogs in the same population can exchange their food information through communication. The frogs in the wetlands are divided into several sub-populations according to a certain strategy and each sub-population has its own culture. The sub-population conducts a local search. When the sub-populations’ local search is satisfied, the information exchange between different sub-populations will begin to complete the global search. The local search and the global search will be conducted alternately until a frog finds the food or the alternating times reach the maximum. Figure 1 shows the process of SFLA. The parameters of SFLA are shown in Table 1.

**Table 1:** The parameters of Shuffled Frog Leaping Algorithm (SFLA).

Number	Parameter	Description
1	*F*	The total number of the frogs in the population
2	*m*	The number of sub-populations
3	*n*	The number of the frogs in a sub-population
4	*P_x_*	The global best frog
5	*P_b_*	The local best frog
6	*P_w_*	The local worst frog
7	*Ls*	The iterations of local search
8	*S_f_*	The iterations of global search
9	*Fitness*	The quality evaluation standard of frog
10	*S_max_*	The frog’s maximum jump step

**Figure 2:**
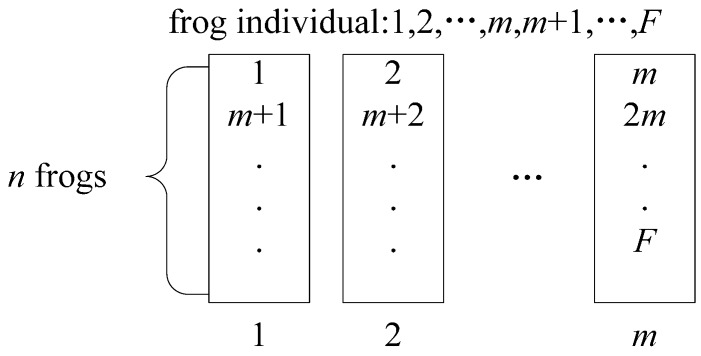
The sub-population division strategy.

In order to balance the search ability of each sub-population, a sub-population division strategy needs to be conducted. The sub-population division strategy is: calculate the fitness value of each frog, sort them in descending (or ascending) order according to the fitness, and finally divide them into sub-populations according to the strategy shown in Figure 2.

The information transferred in the sub-population is realized through the influence of *P_b_* on *P_w_*. It is given by:

(1) Calculate the jump step of *P_w_*
  $$S=\min\{\mathrm{int}[rand\times (P_{b}-P_{w})],S_{\text{max}}\},\text{for a positive step}$$
  (4) $$S=\max\{\mathrm{int}[rand\times (P_{b}-P_{w})],-S_{\text{max}}\},\text{for a negative step}$$
(2) Evolve *P_w_*
  (5) $$P_{w}\prime =P_{w}+S$$

GA, ACO, PSO, MA and SFLA are all meta-heuristics. GA begins with numbers of stochastic individuals and searches the solution space through mutation and crossover between individuals. ACO is a probability-type algorithm to construct an optimal path in a graph. It begins with numbers of empty individuals and gradually constructs them by randomly choosing paths according to their pheromone; a parameter indicates the quality of each path. PSO begins with numbers of stochastic individuals just like a genetic algorithm, but it finds the global optimal solution by iteratively following the best solution it has found ever before. MA is a combination of population-based global search and individual-based local heuristic search. SFLA combines population-based global search and sub-population-based local search. During the local search stage, SFLA moves on by the influence of its local and global best solutions.

Note that SFLA combines the search strategy of PSO and MA. It conducts population-based global search like MA and sub-population-based local search by iteratively following the local and global best solutions like PSO. Therefore, SFLA has both the benefits of PSO and MA. Compared with GA, SFLA moves to the aim solution more quickly because it has higher search directivity.

As a swarm intelligent optimization algorithm, SFLA combines the advantages of the Particle Swarm Optimization and the Memetical Algorithm. It is simple and easy to realize. It has fewer parameters, fast convergence speed and a strong global search capability. The SFLA mainly focuses on continuous optimization problems, so it has fewer outcomes in the discrete combinatorial optimization problems. In [13], a discrete SFLA is proposed to solve the Traveling Salesman Problem, but SFLA’s application in real-time task scheduling problems cannot be found currently.

## 5. Applying SFLA to *e-RTSP*

In this section, SFLA is applied to solve the energy-aware real-time task scheduling on HMS. As SFLA is originally designed for continuous optimization problems, while *e*-*RTSP* is a discrete optimization problem, thus our main aim is to redesign SFLA and apply it to discrete optimization problem. Also, the following optimization procedures are proposed to quickly obtain the feasible solution with the minimal energy consumption. First, the encoding scheme and the fitness function are defined. Then, the information transfer mode between frogs is designed. Next, the local and global search strategies of SFLA are applied to schedule real-time tasks. Finally, a novel optimization scheme is presented to avoid the premature and local optimal solution.

### 5.1. Encoding

Encoding is the first step to modify SFLA for *e*-*RTSP*, a discrete optimization problem. An encoding scheme is designed and it maps a scheduling scheme to a frog of SFLA. The real-time task-scheduling scheme mapped to a frog is realized by compressing the scheduling matrix *X_n×k_*. The compression method is shown as follows: remove the elements with value 0, retain the elements with value 1 and replace these elements with their column numbers, finally a one-dimensional array $Frog=\{P_{1i},P_{2j},...,P_{nl}\}$ (where *i*, *j*, *l* = 1, 2, …, *k*, *i ≠ j ≠ l*, *k* is the number of processors , *n* is the number of tasks) is attained. In the one-dimensional array, the index denotes the task number and the element denotes the processor number the task is assigned to. It can be seen that each frog is a solution of the scheduling problem. According to whether the frog satisfies the constraints (4), it may have two states, namely feasible or infeasible. The feasible frog is the target of SFLA.

Table 2 shows a scheduling matrix of assigning 10 tasks to four processors. The corresponding encoding frog is {2,1,2,0,3,3,0,1,3,0}.

**Table 2:** Scheduling matrix *X_10×4_*.

	*Processors*	*P*_0_	*P*_1_	*P*_2_	*P*_3_
*Tasks*					
*T*_0_		0	0	1	0
*T*_1_		0	1	0	0
*T*_2_		0	0	1	0
*T*_3_		1	0	0	0
*T*_4_		0	0	0	1
*T*_5_		0	0	0	1
*T*_6_		1	0	0	0
*T*_7_		0	1	0	0
*T*_8_		0	0	0	1
*T*_9_		1	0	0	0

### 5.2. Fitness Function

Defining the fitness function is the second step to modify SFLA for *e*-*RTSP*. In the original SFLA, the fitness function is usually a consecutive function, which is not suitable for *e*-*RTSP*. However, a feasible choice to define the fitness function is to seek information from the effect of the fitness function. The fitness function is a specific type of objective function, which is used to summarize how close a given scheduling solution is to achieving the optimal aim. In *e*-*RTSP*, the goal is to quickly find a scheme, which is schedulable and consumes less energy. Therefore, the fitness function is designed as classical fitness and energy-aware fitness, respectively.

As for the classical real-time scheduling problem, the fitness function is defined to optimize the number of feasible solutions, which is shown as follow:

(6) Fitness=nSchedulable

where *nSchedulable* is the number of the processors whose assigned computing capacity have not exceeded its maximum computing capacity.

However, the aim of *e*-*RTSP* is to minimize the energy consumption. The energy-aware fitness is defined as *Fitness_Energy*, whose calculation equation is:

(7) Fitness_Energy=Energy/Max_Energy

where the definitions of *Energy* and *Max_Energy* are from Equations (2) and (3).

### 5.3. Information Transfer Modes

Designing the information transfer mode is the third step as well as the most important step to modify SFLA for *e*-*RTSP*. In this step, the core of the original SFLA is modified to realize the search in the solution space of *e*-*RTSP*. It can be seen from Equations (4) and (5) that when SFLA is applied to solve discrete combinatorial optimization problems, the information transfer mode between frogs should be changed flexibly. According to the basic idea of SFLA and Equations (4) and (5), it can be seen that the essence of SFLA’s information transfer is that the high fitness individual influences the low fitness individual’s mind. From this perspective, two kinds of discrete information transfer modes are designed.

(1) Divergent Information Transfer

A one-dimensional array *R*[*n*] is used to realize the divergent information transfer. Every element of *R*[*n*] is calculated by rand()%2. If *R*[*i*] = 1, replace *P_w_*’s current element with *P_b_*’s current element. *S*_max_ denotes the maximum number of the replacement elements between two frogs. Whether to set up *S*_max_ or not depends on the algorithm’s performance. We take a scheduling problem with 10 tasks and four processors as an example. In a sub-population, *P_b_* = {1,0,0,3,2,0,1,2,1,3} and *P_w_* = {0,1,0,1,3,2,2,3,1,0}. If *R*[*n*] = {1,0,0,1,1,0,1,0,0,0}, then the *P_w_*’ = {1,1,0,3,2,2,1,3,1,0}.

(2) Concentrated Information Transfer

An *inherited culture ration* λ and a starting point *r* are used to realize the concentrated information transfer. *r* is in [0,⌊n×(1−λ)⌋), where *n* is the number of the tasks. *S*_max_ can be described as $S_{\max}=\left\lfloor \text{λ}\times n\right\rfloor$. The concentrate information transfer can be realized by replacing *P_w_*’s elements with *P_b_*’s elements from index *r* to (*r* + *S*_max_ − 1). We also take a scheduling problem with 10 tasks and four processors as an example. In a sub-population, *P_b_* = {1,0,0,3,2,0,1,2,1,3} and *P_w_* = {0,1,0,1,3,2,2,3,1,0}. If λ = 0.40 and *r* = 3, then *S*_max_ = 4 and *P_w_*’ = {0,1,0,3,2,0,1,3,1,0}.

These two information transfer modes both realize the information exchange between *P_b_* and *P_w_*. SFLA using a divergent information transfer mode is called *D-SFLA* and that using a concentrate information transfer mode is called *C-SFLA.*

### 5.4. SFLA Algorithm for e-RTSP

Having modified for discrete optimization problem *e*-*RTSP*, SFLA is ready to be applied to assign real-time tasks to heterogeneous processors. First, the whole process of applying SFLA to *e*-*RTSP* is given. Next, the sub-population information transfer process of applying SFLA to *e*-*RTSP* is described in detail.

(1) Applying SFLA’s Whole Process

According to the basic idea of SFLA, the whole process of applying SFLA to assign real-time tasks to heterogeneous processors is shown as follows:

**Step** **1** Initialize parameters. Initialize the number of scheduling schemes *F* in the population, the number of sub-populations *m*, the number of the scheduling schemes *n* in each sub-population. Initialize the iterations of the sub-population and the maximum iterations when *P_x_* cannot be improved. Initialize the parameters of *e*-*RTSP*, include the number of tasks, the number of processors, the heterogeneity of tasks, processors and the utilization matrix.
**Step** **2** Generate the original scheduling scheme set. Generate *F* scheduling schemes randomly in the solution space, namely generating *F* frogs.
**Step** **3** Divide the scheduling scheme set into *m* sub-populations. Calculate the fitness of each frog and sort them in descending order according to their fitness. Divide them into *m* sub-populations. Determine *P_x_* of the population, *P_b_* and *P_w_* of each sub-population.
**Step** **4** Transfer information in each sub-population. Conduct the information transfer mode in each sub-population according to the iterations of the sub-population. The information transfer process searches the solution space of the scheduling problem and the frogs will jump to the better scheduling schemes.
**Step** **5** Transfer information between sub-populations. Combine the frogs in each sub-population back to the population and sort them in descending order according to their fitness. Update *P_x_*.
**Step** **6** Check the termination conditions. If SFLA finds a feasible scheduling scheme or the iterations where *P_x_* cannot be improved to reach the maximum, then stop the operation of SFLA. Else return to Step 3.

(2) Information Transfer Process in Sub-Populations

In the whole process of SFLA, Step 4 is the core. To ensure that the frogs to jump towards the feasible scheduling schemes, the information transfer in each sub-population needs to obey a certain rule. The information transfer rule in each sub-population is: transfer information from *P_b_* to *P_w_*, and obtain a *P_w_*’. If the fitness of *P_w_*’ is better than that of *P_w_*, replace *P_w_* with *P_w_*’. Else replace *P_b_* with *P_x_* to transfer information to *P_w_* and obtain a new *P_w_*’. If the fitness of *P_w_*’ is better than that of *P_w_*, replace *P_w_* with *P_w_*’. Else generate a new frog randomly and replace *P_w_* with the new frog. The pseudo code of the information transfer process in sub-population is shown in Algorithm 1.

**Algorithm 1. Sub-Population Information Transfer Algorithm.**


Input: an original scheduling scheme set.
Output: an updated scheduling scheme set.
1. **Determine** *P_x_*;
2. **for** each memeplex *i*{
3.   **for** each iteration *j*{
4.      **Determine** *P_b_*, *P_w_*;
5.      *P_w_*’ = *P_w_* applies Equations (4) and (5) with *P_b_*;
6.      Evaluate the fitness of *P_w_*’;
7.      **if** (*P_w_*’ is better than *P_w_*)
8.         Replace *P_w_* with *P_w_*’;
9.      **else**{
10.     *P_w_*’ = *P_w_* applies Equations (4) and (5) with *P_x_*;
11.        Evaluate the fitness of *P_w_*’;
12.        **if** (*P_w_*’ is better than *P_w_*)
13.           Replace *P_w_* with *P_w_*’;
14.        **else**{
15.           Generate a new frog *P_w_*’;
16.           Evaluate the fitness of *P_w_*’;
17.           Replace *P_w_* with *P_w_*’;}}}}

For a sub-population, *P_x_* and *P_b_* influence *P_w_* alternately, leading the frogs to jump towards the feasible scheduling schemes. If *P_w_* cannot be improved, a new frog is generated to replace it to ensure the activeness of the population.

### 5.5. Precocity Remission

One of SFLA’s drawbacks applied to *e*-*RTSP* is *precocity*. With little diversity in the population, SFLA may become less improvable before it has fully searched the solution space of the *e*-*RTSP* problem. This phenomenon is called *precocity*. In the process of the sub-population information transfer, Equations (4) and (5) are applied to *P_w_* frequently, leaving the structure of *P_w_* similar to that of *P_x_* and *P_b_*. We imported an algorithm to remit the precocity. Its pseudo code taking *P_x_* for example is shown in Algorithm 2.

In Algorithm 2, the structure of *P_x_* is disturbed without reducing its fitness. The diversity of the whole population is guaranteed by applying Algorithm 2. *P_b_* is disturbed after every information transfer iteration in the sub-population and *P_x_* is disturbed after every information transfer iteration between sub-populations. Thus, it will not add much computing overhead to SFLA.

**Algorithm 2. *P_x_* Disturbance Algorithm.**


Input: *P_x_*.
Output: a new *P_x_* whose structure is disturbed.
1. **for** each task *i*{
2.   **for** each processor *j*{
3.     *t* = the number of the processor that task *i* is assigned in *P_x_*;
4.      **if** (*j* is equal to *t*)
5.         Go to Line 2;
6.      **else**{
7.         *temp*_*solution* = *P_x_* replaces *t* with *j*;
8.         Evaluate the fitness of *temp*_*solution*;
9.         **if** (the fitness of *temp*_*solution* is larger than that of *P_x_*)
10.           *P_x_* = *temp*_*solution*;}}}

### 5.6. Local Optimal Avoidance

Another drawback of SFLA applied to *e*-*RTSP* is that it is easy to fall into a local optimal solution. A local optimal solution is a scheme in which tasks are assigned to processors extremely unevenly. Namely the fitness of the local optimal solution is quite high, but the assigned computing capacity of the processor exceeds its maximal computing capacity. Construction of a sub-population in each sub-population can prevent the occurrence of such a situation to a certain extent. To prevent local optimal solutions, several frogs in a sub-population will be selected into the sub-population. As the convergence speed of SFLA cannot be slowed down, the frogs with higher fitness should be assigned a higher selected probability. As in [13,14,15,16,17,18,19,20,21,22,23,24,25,26,27,28,29,30,31,32], the selected probability of each frog is defined as follows:

(8) $$p\text{(}j\text{)}=\frac{2(n+1-j)}{n(n+1)},j=\text{ 1},\text{2},\;\ldots ,n$$

where *n* is the number of the frogs in a sub-population.

As the frogs in a sub-population are sorted in descending order according to their fitness, so Equation (8) gives higher selected probability to the frogs with higher fitness.

### 5.7. Convergence Acceleration

As the original population is generated randomly when applying SFLA’s whole process to *e*-*RTSP*, the overall qualities of these scheduling schemes are low. We apply the neighborhood search to the original population to improve the fitness of each frog. It consists of two parts: (1) migrate a task from a processor to a different one if the energy consumption becomes less without lowering the fitness; and (2) exchange two tasks between two processors if the energy consumption becomes less without lowering the fitness. A frog becomes a better solution in its neighborhood by applying the neighborhood search. The overall quality of the population is improved with this preprocessing and so SFLA can find a feasible scheduling scheme more quickly.

### 5.8. Summary of the SFLA Applied to e-RTSP

**Figure 3:**
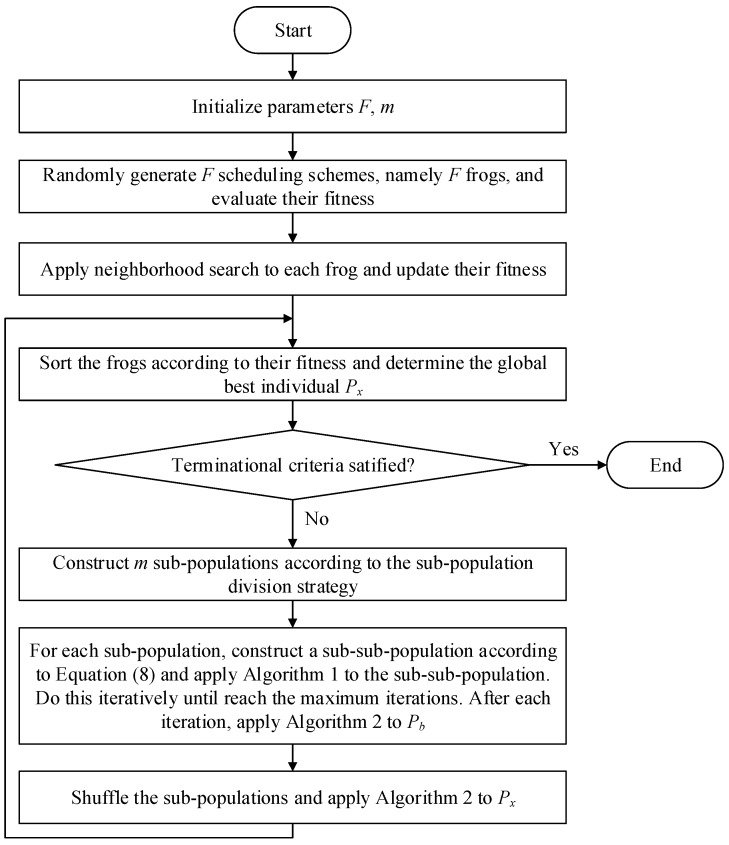
Flow chart of SFLA for *e*-*RTSP.*

In this section, Figure 3 concludes the above procedures.

## 6. Experiment

Extensive experiments are conducted to estimate SFLA’s performance for the energy-aware real-time task-scheduling problem. Our task and processor sets are not only generated from *synthetic* data for simulation but also from *real* data of benchmarks. The *synthetic* data are generated following the same setting in [11]. The *real* data are from the Embedded System Synthesis Benchmarks Suite (E3S) [37].

First, we generate the synthetic data sets for simulation. Then, the parameter tests are conducted to determine the optimal parameters for SFLA. After that, SFLA is compared with several familiar scheduling algorithms with *synthetic* and *real* data of benchmarks. Their performance is analyzed in detail.

### 6.1. Generation of Synthetic Data Sets for Simulation

First, the generating method of the utilization matrix is introduced. φ*_P_* denotes the heterogeneity of multiprocessors *P* and φ*_T_* denotes the heterogeneity of real-time tasks *T*. To make the experiment more practical, the utilization matrix must reflect φ*_P_* and φ*_T_*. The utilization matrix can be generated by the following steps, where *n* is the number of tasks, *m* is the number of processors, *i* is in the interval [1, *n*] and *j* is in the interval [1, *m*].

**Step** **1** Generate a one-dimensional integer array *cycle*[*n*] randomly. Each *cycle*[*i*] is in [100, 1000] and is equal to the number of the clock cycles that *T_i_* needs.
**Step** **2** Generate a one-dimensional floating-point array *T_B_*[*n*] randomly. Each *T_B_*[*i*] is in [1, φ*_T_*]. It is a task baseline vector.
**Step** **3** Calculate the one-dimensional array *period*[*n*]. *period*[*i*] is the period of *T_i_* and is calculated from *cycle*[*i*]/*T_B_*[*i*].
**Step** **4** Generate a two-dimensional integer array *speed*[*n*][*m*] randomly. For each *T_i_*, generate a one-dimensional array *speed*[*i*][*m*]. Each *speed*[*i*][*j*] is limited in [φ*_T_*, φ*_T_* ×φ*_P_*].
**Step** **5** Calculate the two-dimensional array *exe*[*n*][*m*]. Each *exe*[*i*][*j*] is equal to the executing time that *T_i_* needs on *P_j_*. The elements are given by *cycle*[*i*]/*speed*[*i*][*j*].
**Step** **6** Calculate the utilization matrix *U_n×m_*. The calculation equation is given by *u_i,j_* = *exe*[*i*][*j*]/*period*[*i*].

Next, the generating method of the test data set is given. A utilization matrix is consistent if a task runs faster on a processor than on the other processors when the other tasks also run faster on them. This test data set contains all 8 combinations of φ*_P_*, φ*_T_* and the consistency, namely Consistent, High φ*_T_*, High φ*_P_*; Consistent, High φ*_T_*, Low φ*_P_*; Consistent, Low φ*_T_*, High φ*_P_*; Consistent, Low φ*_T_*, Low φ*_P_*; Inconsistent, High φ*_T_*, High φ*_P_*; Inconsistent, High φ*_T_*, Low φ*_P_*; Inconsistent, Low φ*_T_*, High φ*_P_*; and Inconsistent, Low φ*_T_*, Low φ*_P_*, where High φ*_T_* is 100, Low φ*_T_* is 5, High φ*_P_* is 20, and Low φ*_P_* is 5. They are, respectively, represented by C_HT_HP, C_HT_LP, C_LT_HP, C_LT_LP, IC_HT_HP, IC_HT_LP, IC_LT_HP, and IC_LT_LP. For each combination 15 test instances are generated, so the SFLA experiment is run on 120 test instances in total. Each test instance runs 10 times. Table 3 shows the utilization matrix scale of SFLA’s parameter test.

**Table 3:** The utilization matrix scale of SFLA’s parameter test.

No.	Config.	Size	No.	Config.	Size
N1	C_HT_HP	U_90×4_	N5	IC_HT_HP	U_140×5_
N2	C_HT_LP	U_50×8_	N6	IC_HT_HP	U_50×8_
N3	C_LT_HP	U_70×4_	N7	IC_LT_HP	U_90×4_
N4	C_LT_LP	U_40×8_	N8	IC_LT_LP	U_50×8_

### 6.2. Parameter Setting for SFLA

Since SFLA is a new combinatorial optimization algorithm, there are few references to determine the values of its parameters. In order to find better parameter values for SFLA, four kinds of parameter tests were conducted. The influences of three factors on D-SFLA’s performance were tested with control variants. They are *the number of the sub-population iterations*, *the sub-populations size* and *the population size*. The *influence of* λ on C-SFLA’s performance was also tested. In the experimental results, *Avg.* stands for the average time that D-SFLA needs to process a problem instance and *Feas*. stands for the times that D-SFLA can find a feasible schedule in the total 150 times running. We also provide the median for each plotted value by running every test for 20 times.

**Figure 4:**
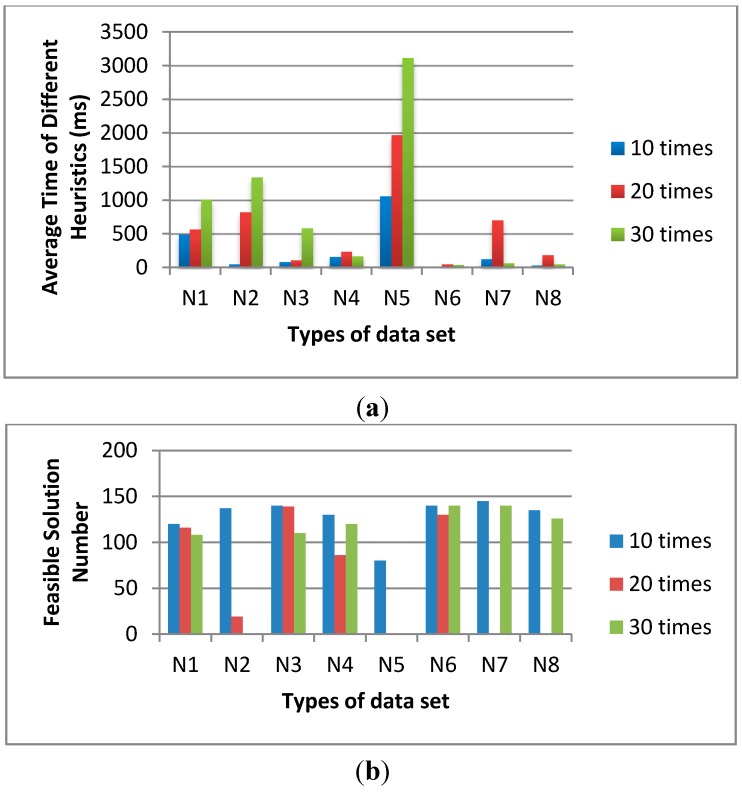
The influence of sub-population iterations on (**a**) average time and (**b**) feasible solution number.

In Figure 4, we fix the population size as 200 and the sub-population size as 20. The iteration number of sub-population is varied from 10, 20 to 30 times. The average time of 10 times is shorter than the others and the feasible solution numbers of 10 times are much more than the others.

**Figure 5:**
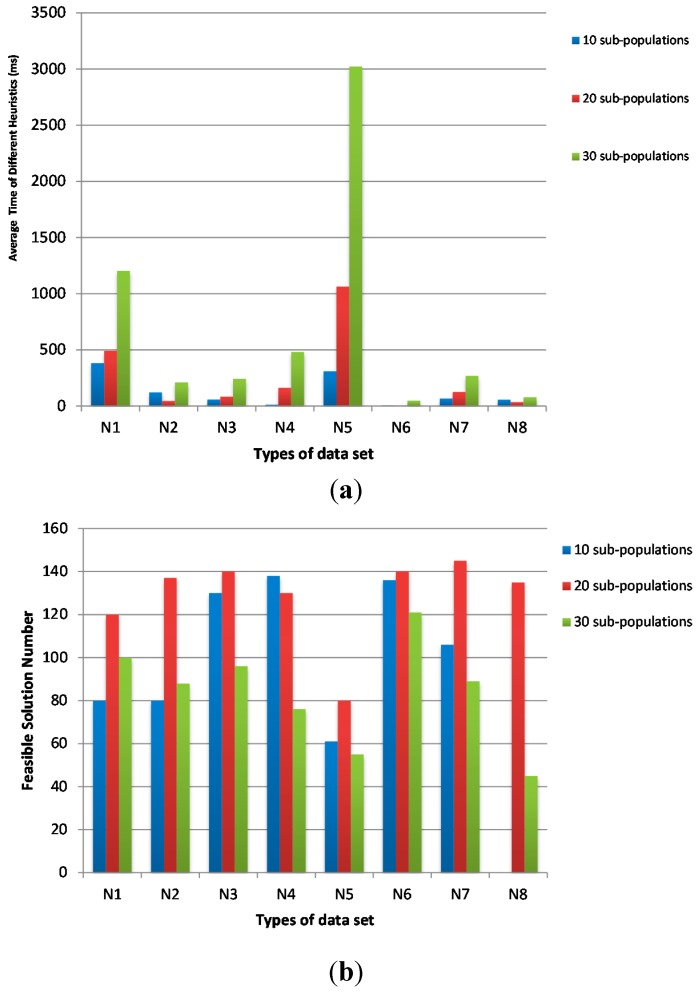
The influence of sub-population size on (**a**) average time and (**b**) feasible solution number.

In Figure 5, we fix the population size as 200 and the sub-population iteration number as 10. The sub-population size is varied from 10, 20 to 30 frogs. The average time of 20 sub-populations is shorter than that of 30 sub-populations and the feasible solution numbers of 20 sub-populations are much higher than that of 30 sub-populations. Although the average time of 10 sub-populations is slightly shorter than that of 20 sub-populations, the feasible solution numbers of 20 sub-populations are much higher than that of 10 sub-populations.

**Figure 6:**
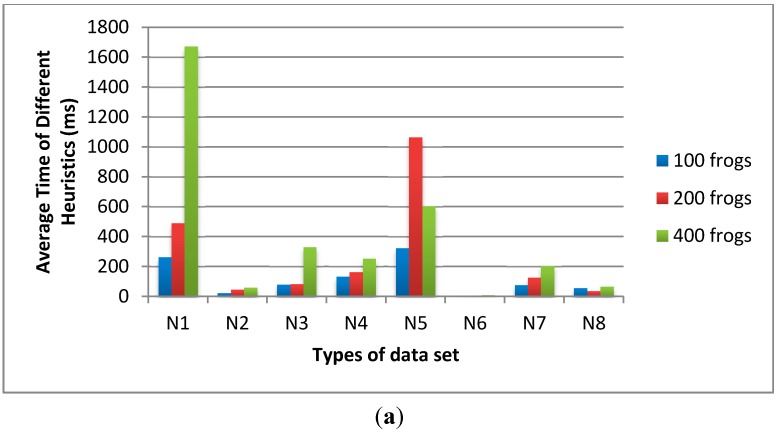

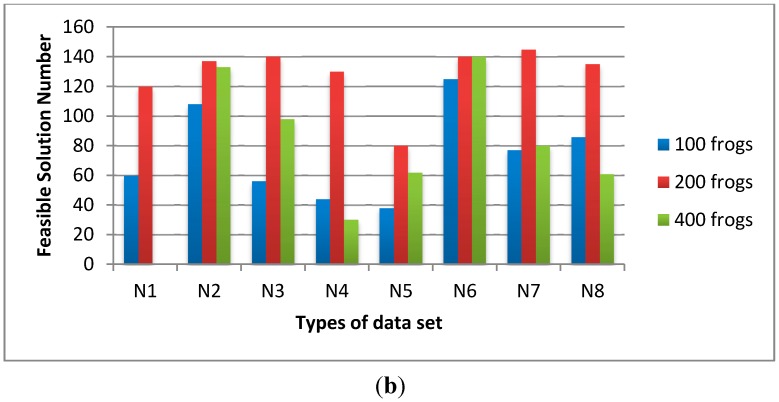
The influence of population size on (**a**) average time and (**b**) feasible solution number.

In Figure 6, we fix the sub-population size as 20 and the iteration number of sub-population as 10 times. The population size is varied from 100, 200 to 400 frogs. The results show that the average time of population size 200 is shorter than the others and the feasible solution numbers of population size 200 are much higher than the others. Therefore, the optimal parameters of D-SFLA are set in Table 4.

**Figure 7:**
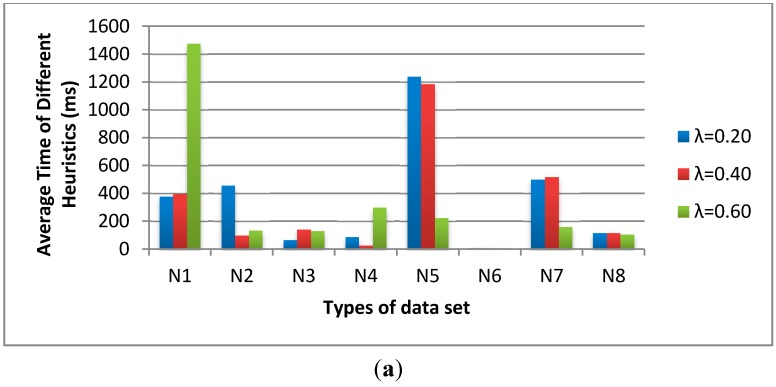

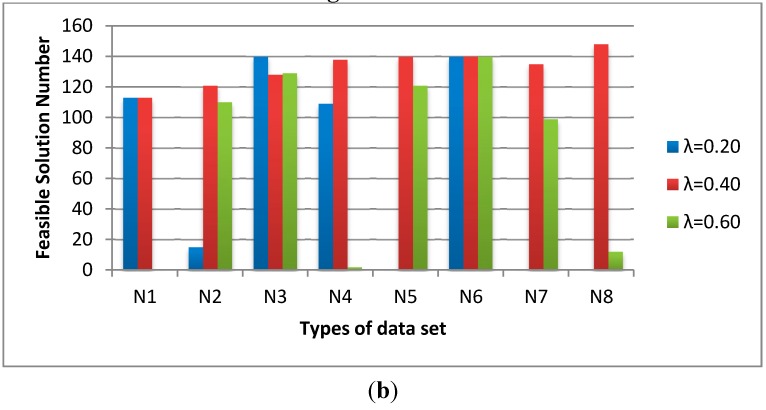
The influence of λ on (**a**) average time and (**b**) feasible solution number.

In Figure 7, we fix the population size as 200, the sub-population size as 20 and the sub-population iteration number as 10. The influence of λ is varied from 0.2, 0.4 to 0.6. The average time of λ = 0.4 is shorter than the others and the feasible solution numbers of λ = 0.4 are much higher than the others. Thus, we determine the λ = 0.4 for the C-SFLA.

**Table 4:** The parameters of SFLA.

No.	Parameter	Value
1	Population Size	200
2	Sub-population Size	20
3	Sub-population Iterations	10

### 6.3. Performance Comparison with Synthetic Data

In order to estimate SFLA’s performance on *e*-*RTSP*, the simulation was implemented in Visual C++ and in a server with a Pentium(R) Dual-Core CPU (E6700@3.20 GHz) and 1 GB memory.

The simulation compares the performance of the algorithms like ACO, GA and ILP. The parameters of GA and ACO for the comparison test are shown in Table 5 and Table 6. In the comparison test, D-SFLA did not set a *S_max_* and C-SFLA set λ to 0.40. The other parameters of D-SFLA and C-SFLA are given in Table 4.

**Table 5:** The parameters of Genetic Algorithm (GA).

No.	Parameter	Value
1	Population Size	200
2	Crossover rate	60%
3	Mutation rate	40%

**Table 6:** The parameters of Ant Colony Optimization (ACO).

No.	Parameter	Value
1	Population Size	10
2	ρ	0.02
3	ω	20.00

Table 7 shows the utilization matrix scale for the algorithms. Section 6.1 describes its generation.

**Table 7:** The utilization matrix scale.

No.	Config.	Size	No.	Config.	Size
N1	C_HT_HP	U_75×4_	N5	IC_HT_HP	U_115×5_
N2	C_HT_LP	U_40×8_	N6	IC_HT_HP	U_55×8_
N3	C_LT_HP	U_60×4_	N7	IC_LT_HP	U_65×4_
N4	C_LT_LP	U_40×8_	N8	IC_LT_LP	U_45×8_

**Figure 8:**
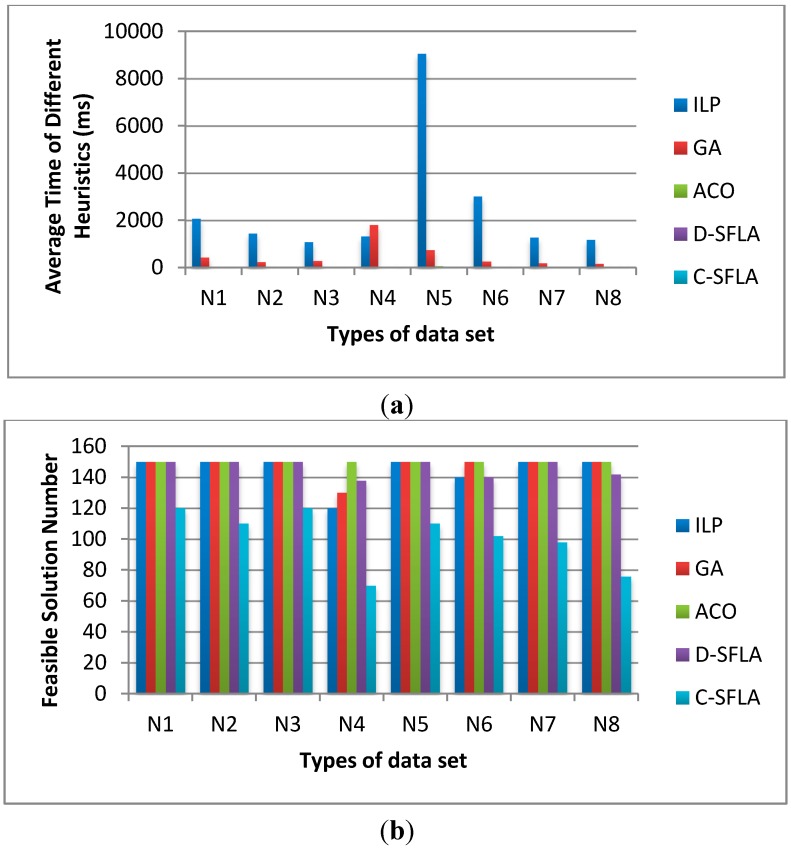
The average runtime and feasible solution numbers of for ILP, GA, ACO, D-SFLA and C-SFLA algorithms: (**a**) average time; and (**b**) feasible solution number.

Figure 8 shows: (1) Average time: ILP is the most time-consuming to acquire feasible solutions, which is nearly 1000 times more than the D-SFLA and C-SFLA algorithms. GA and ACO also cost more time than D-SFLA and C-SFLA, which are 200 times and 20 times more, respectively, than the SFLA ones. The D-SFLA and C-SFLA algorithms can acquire the feasible solution in less than 20 ms in all the conditions. (2) Feasible solution number: The ACO algorithm can acquire all the 150 solutions, which has the best performance. The feasible solution numbers of ILP, GA and D-SFLA are almost the same, which is 7% less than that of the ACO algorithm. However, the feasible solution number of the C-SFLA algorithm is 20% less than that of the other algorithms. Thus, we choose the GA, ACO and D-SFLA to compare the energy consumption.

**Figure 9:**
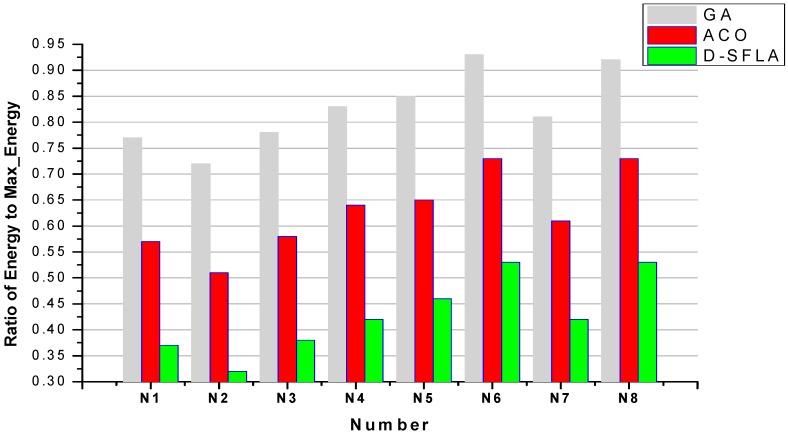
The energy consumption of GA, ACO and D-SFLA for *e*-*RTSP*.

Figure 9 shows the energy consumption of the solutions provided by GA, ACO and D-SFLA algorithms for *e*-*RTSP*. According to Equation (1), energy consumption *E_i,j_* is determined by the clock frequency *f_j_* and the number of clock cycles *cycle_i_* of *T_i_*. Thus, energy consumption of each feasible solution can be calculated based on Equations (1)–(3). The metric is the ratio of the *Energy* to *Max_energy* of a feasible solution, which is defined in Equations (2) and (3). We can see that D-SFLA uses 30% and 60% less energy than ACO and GA algorithms, respectively.

### 6.4. Performance Comparison with Real Data from Benchmarks

In the experiments, the Embedded System Synthesis Benchmarks Suite (E3S) is used [37]. E3S is built based on the Embedded Microprocessor Benchmark Consortium (EEMBC) benchmarks suite [38], which has 20 kinds of processors. Each processor has records of real data with code sizes, speeds and task execution times. There are five kinds of benchmarks: Automotive/Industrial Benchmarks (16 tasks), Networking Benchmarks (5 tasks), Consumer Benchmarks (5 tasks), Office Automation Benchmarks (3 tasks) and Telecomm Benchmarks (16 tasks). E3S also gives the parameters of a task. In our experiments, we choose the following four kinds of processors: AMD ElanSC520-133 MHz, AMD K6-2E 400 Mhz/ACR, AMD K6-2E+ 500 Mhz/ACR and AMD K6-IIIE+ 550 Mhz/ACR because the code size of each task on these processors is the same.

**Figure 10:**
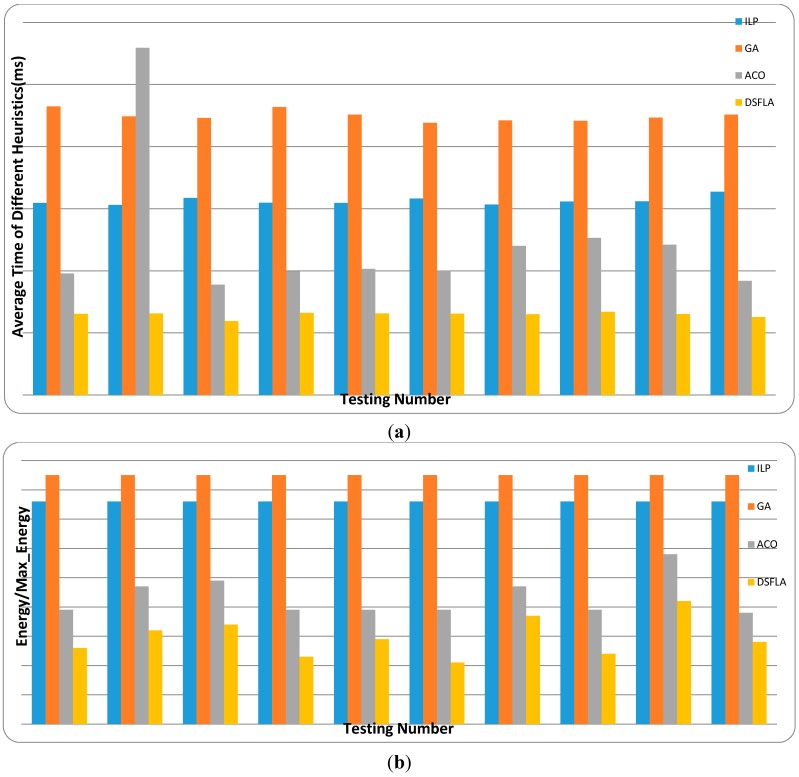
The average runtime and energy consumption of real data set 1 for different heuristics: (**a**) average time and (**b**) energy consumption.

**Figure 11:**
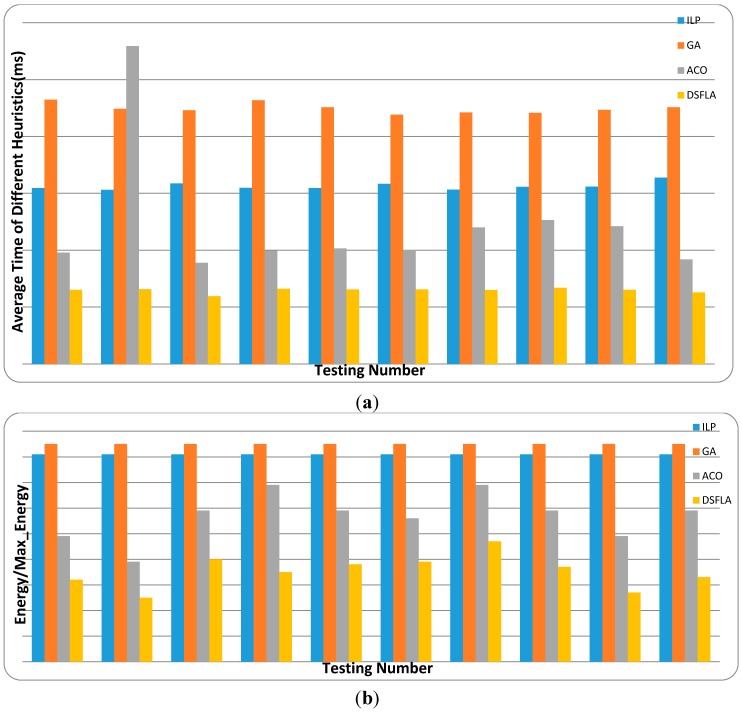
The average runtime and energy consumption of real data set 2 for different heuristics: (**a**) average time and (**b**) energy consumption.

In our experiments, we assign the 46 tasks to the four processors. The number of clock cycles for each task can be calculated by the product of the processor speed and task execution time. We determine the deadline for each task in regarding to the schedulability of the problem. According to the structure of the heterogeneous platform, two kinds of test data sets (Real Data Set 1 and Real Data Set 2) can be picked up from E3S. For each kind of test data set, we run the four algorithms (ILP, GA, ACO, and D-SFLA) 10 times. The parameters of the four algorithms are set as shown in Table 4, Table 5 and Table 6. The results are shown in Figure 10 and Figure 11.

Note that ILP does not obtain the optimal solution for the energy consumption. The reason is shown as follows: The feasibility of the scheduling scheme is the primary goal of the real-time task scheduling algorithm and then the optimization of energy consumption. As is well-known, if the linear programming algorithm finds a feasible solution, then this solution must be an optimal solution. Therefore, if the objective function of linear programming is to minimize the energy consumption, then the computing capacity of each processor will be occupied completely in the solution. In that way, the tasks divided and assigned to more than one processor in the solution won’t be able to be assigned again, which means that the linear programming algorithm with this objective function cannot find a feasible solution. Considering this fact, when applying the linear programming algorithm to our real-time task-scheduling problem, we hold the primary goal and give the second up. So we set the objective function of linear programming as minimizing the computing consumption. Obviously, this objective function will make the solution with the highest energy consumption. On the other hand, every task in our problem model is forbidden to be divided, so the linear programming algorithm is modified to deal with integer variable problem by assigning the tasks divided and assigned to more than one processor in the solution again. The modified linear programming algorithm is called integer linear programming (ILP). Therefore, the solution of ILP may be not the solution with the highest energy consumption. This also gives an explanation of the reason why the performance of ILP in energy optimization is better than GA, but worse than ACO and D-SFLA.

## 7. Conclusions

The Energy-Aware Real-Time Tasks Scheduling Problem for heterogeneous processor systems named *e-RTSP* is formulated as a combinatorial optimization problem. A new meta-heuristic algorithm for real-time task scheduling based on the SFLA paradigm is presented, not only satisfying the task deadlines but also reducing the energy costs. An extensive experiment shows that this algorithm has much better performance than GA and ACO approximation algorithms. The D-SFLA algorithm provides solutions, which use 30% and 60% less energy than those given by the ACO and GA algorithms. Also, the time of D-SFLA for the feasible solutions is 20 and 200 times less than ACO and GA, respectively.

Current research mainly focuses on modeling independent and periodic real-time applications. However, more complicated and irregular applications should be considered. In the future, we plan to relax our restrictions on the task set by considering task precedence and inter-task communication. Also, our energy consumption model puts emphasis on the energy consumption of CPUs. The additional energy consumption of the memory access and I/Os will be investigated in future.

## References

[1] Hamutal M. (2006). Power and Energy-Aware Architectural Techniques for High-Performance Uniprocessor and Multiprocessor Systems. Ph.D. Thesis.

[2] Kumar R., Tullsen D.M., Jouppi N.P. Core architecture optimization for heterogeneous chip multiprocessors. Proceedings of the 15th International Conference on Parallel Architectures and Compilation Techniques.

[3] Chantem T., Hu X.S., Dick R.P. (2011). Temperature-Aware scheduling and assignment for hard real-time applications on MPSoCs. IEEE Trans. Very Large Scale Integr. (VLSI) Syst..

[4] Lin J., Cheng A.M.K., Kumar R. Real-Time task assignment in heterogeneous distributed systems with rechargeable batteries. Proceedings of the International Conference on Advanced Information Networking and Applications, AINA’09.

[5] Baruah S. Feasibility analysis of preemptive real-time systems upon heterogeneous multiprocessor platforms. Proceedings of the 25th IEEE International on Real-Time Systems Symposium.

[6] Yu Y., Prasanna V.K. Power-Aware resource allocation for independent tasks in heterogeneous real-time systems. Proceedings of the Ninth International Conference on Parallel and Distributed Systems.

[7] Chen J.J., Schranzhofer A., Thiele L. Energy minimization for periodic real-time tasks on heterogeneous processing units. Proceedings of the IEEE International Symposium on Parallel & Distributed Processing, IPDPS 2009.

[8] Chen J.J., Kuo T.-W. Allocation cost minimization for periodic hard real-time tasks in energy-constrained DVS systems. Proceedings of the 2006 IEEE/ACM International Conference on Computer-Aided Design.

[9] Hung C.-M., Chen J.-J., Kuo T.-W. Energy-Efficient real-time task scheduling for a DVS system with a non-DVS processing element. Proceedings of the 27th IEEE International Real-Time Systems Symposium, RTSS’06.

[10] Yang C.-Y., Chen J.-J., Kuo T.-W., Thiele L. (2009). An approximation scheme for energy-efficient scheduling of real-time tasks in heterogeneous multiprocessor systems. Proceedings of the Conference on Design, Automation and Test in Europe.

[11] Chen H., Cheng A.M.K., Kuo Y.-W. (2011). Assigning real-time tasks to heterogeneous processors by applying ant colony optimization. J. Parallel Distrib. Comput..

[12] Eusuff M.M., Lansey K.E. (2003). Optimization of water distribution network design using the shuffled frog leaping algorithm. J. Water Resour. Plan. Manag..

[13] Luo X.-H., Yang Y., Li X. Solving TSP with shuffled frog-leaping algorithm. Proceedings of the Eighth International Conference on Intelligent Systems Design and Applications, ISDA’08.

[14] Cheng A.M.K. (2003). Real-Time Systems: Scheduling, Analysis, and Verification.

[15] Davis R.I., Burns A. (2011). A survey of hard real-time scheduling for multiprocessor systems. ACM Comput. Surv. (CSUR).

[16] Baruah S.K. Partitioning real-time tasks among heterogeneous multiprocessors. Proceedings of the International Conference on Parallel Processing, ICPP 2004.

[17] Baruah S.K. Task Partitioning upon Heterogeneous Multiprocessor Platforms. Proceedings of the IEEE Real-Time and Embedded Technology and Applications Symposium.

[18] Chen H., Cheng A.M.K. (2005). Applying ant colony optimization to the partitioned scheduling problem for heterogeneous multiprocessors. ACM SIGBED Rev..

[19] Lin X., Lu Y., Deogun J., Goddard S. Real-Time divisible load scheduling for cluster computing. Proceedings of the 13th IEEE Real Time and Embedded Technology and Applications Symposium, RTAS’07.

[20] Lin X., Lu Y., Deogun J., Goddard S. Real-time divisible load scheduling with different processor available times. Proceedings of the International Conference on Parallel Processing, ICPP 2007.

[21] Bharadwaj V., Ghose D., Robertazzi T.G. (2003). Divisible load theory: A new paradigm for load scheduling in distributed systems. Clust. Comput..

[22] Wiese A., Bonifaci V., Baruah S. (2013). Partitioned EDF scheduling on a few types of unrelated multiprocessors. Real-Time Syst..

[23] Andersson B., Raravi G., Bletsas K. Assigning real-time tasks on heterogeneous multiprocessors with two unrelated types of processors. Proceedings of the 2010 IEEE 31st Real-Time Systems Symposium (RTSS).

[24] Stavrinides G.L., Karatza H.D. (2012). Scheduling real-time DAGs in heterogeneous clusters by combining imprecise computations and bin packing techniques for the exploitation of schedule holes. Future Gener. Comput. Syst..

[25] Qin X., Jiang H. (2005). A dynamic and reliability-driven scheduling algorithm for parallel real-time jobs executing on heterogeneous clusters. J. Parallel Distrib. Comput..

[26] Kianzad V., Bhattacharyya S.S. (2006). Efficient techniques for clustering and scheduling onto embedded multiprocessors. IEEE Trans. Parallel Distrib. Syst..

[27] Auluck N., Agrawal D.P. A scalable task duplication based algorithm for improving the schedulability of real-time heterogeneous multiprocessor systems. Proceedings of the 2003 International Conference on Parallel Processing Workshops.

[28] Yoo M., Gen M. (2007). Scheduling algorithm for real-time tasks using multiobjective hybrid genetic algorithm in heterogeneous multiprocessors system. Comput. Oper. Res..

[29] Yoo M. (2009). Real-Time task scheduling by multiobjective genetic algorithm. J. Syst. Softw..

[30] Yang C.Y., Chen J.-J., Kuo T.-W., Thiele L. Energy reduction techniques for systems with non-DVS components. Proceedings of the IEEE Conference on Emerging Technologies & Factory Automation, ETFA 2009.

[31] Li D., Wu J. (2012). Energy-Aware Scheduling on Multiprocessor Platforms.

[32] Schmitz M.T., Al-Hashimi B.M., Eles P. Energy-Efficient mapping and scheduling for DVS enabled distributed embedded systems. Proceedings of the Design, Automation and Test in Europe Conference and Exhibition.

[33] Luo J., Jha N.K. Static and dynamic variable voltage scheduling algorithms for real-time heterogeneous distributed embedded systems. Proceedings of the 2002 Asia and South Pacific Design Automation Conference.

[34] Burd T.D., Brodersen R.W. Energy efficient CMOS microprocessor design. Proceedings of the Twenty-Eighth Hawaii International Conference on System Sciences.

[35] Zhu D., Melhem R., Childers B.R. (2003). Scheduling with dynamic voltage/speed adjustment using slack reclamation in multiprocessor real-time systems. IEEE Trans. Parallel Distrib. Syst..

[36] Liu C.L., Layland J.W. (1973). Scheduling algorithms for multiprogramming in a hard-real-time environment. J. ACM (JACM).

[37] Embedded System Synthesis Benchmarks Suite (E3S). http://ziyang.eecs.umich.edu/~dickrp/e3s/.

[38] Embedded Microprocessor Benchmark Consortium http://www.eembc.org.

